# Simplicial Persistence of Financial Markets: Filtering, Generative Processes and Structural Risk

**DOI:** 10.3390/e24101482

**Published:** 2022-10-18

**Authors:** Jeremy Turiel, Paolo Barucca, Tomaso Aste

**Affiliations:** 1 Department of Computer Science, UCL, Gower Street, London WC1E 6BT, UK; 2 JP Morgan, 60 Victoria Embankment, London EC4Y 0JP, UK

**Keywords:** network theory, topological filtering, network motif, motif persistence, long memory, complex systems, time series analysis, financial networks

## Abstract

We introduce simplicial persistence, a measure of time evolution of motifs in networks obtained from correlation filtering. We observe long memory in the evolution of structures, with a two power law decay regimes in the number of persistent simplicial complexes. Null models of the underlying time series are tested to investigate properties of the generative process and its evolutional constraints. Networks are generated with both a topological embedding network filtering technique called TMFG and by thresholding, showing that the TMFG method identifies high order structures throughout the market sample, where thresholding methods fail. The decay exponents of these long memory processes are used to characterise financial markets based on their efficiency and liquidity. We find that more liquid markets tend to have a slower persistence decay. This appears to be in contrast with the common understanding that efficient markets are more random. We argue that they are indeed less predictable for what concerns the dynamics of each single variable but they are more predictable for what concerns the collective evolution of the variables. This could imply higher fragility to systemic shocks.

## 1. Introduction

Networks representing the structure of the interactions within complex systems have been increasingly studied in the last few decades [1]. Applications range from biological networks to social networks, infrastructures and finance [2]. In finance—mainly due to the abundance of time-series data regarding economic entities and the lack of data on direct relationships between them—there has been an extensive focus on the estimation of interactions between financial variables from cross-correlations [3]. The need to extract significant links from noisy correlation matrices has triggered the development of filtering techniques which yield significant sparse network structures [4,5,6,7] based on a limited set of statistical or topological hypotheses.

There are three main approaches to network filtering: (i) thresholding, (ii) statistical validation, and (iii) topological filtering. These methods have demonstrated that a meaningful and consistent taxonomy of financial assets emerges from sparse network structures [3,8,9,10]. Thresholding methods remove edges which are less significant based on their strength (or its absolute value); quantile thresholding is one of these methods, which we use in this paper. This method considers the distribution of edge strengths and removes edges with strength below a certain quantile level. It is often applied to financial correlation matrices due to its lack of assumptions on the underlying distribution. Statistical validation—which constitutes a generalisation of simpler thresholding methods—has been used to establish the significance of edges in correlation matrices, with applications to economics and finance as well as to other fields [11,12,13,14,15]. Statistical validation can be implemented by comparing the empirical correlations with null-hypothesis correlations generated from time-series randomized over the time dimension to remove dependency. Topological filtering was initially suggested by Mantegna [3] by means of the Minimum Spanning Tree (MST) technique and then it was extended to planar graphs with the Planar Maximally Filtered Graph (PMFG) [16] and more recently to chordal graphs with predefined motif structure, as the Triangulated Maximally Filtered Graphs (TMFG) in [17] and the Maximally Filtered Clique Forest (MCFC) in [18]. In the present work we make use of the TMFG which is constructed from the correlation matrix by starting from the maximal weight tetrahedron, then it proceeds by adding vertices within triangular faces iteratively so that the total weight is maximised. This generates a planar graph which is a tree composed of tetrahedral cliques. This algorithm is computationally highly efficient.

In this paper, we make use of filtering networks to investigate the temporal evolution of the market structure and we compare the persistence of certain network motifs with different levels of market efficiency. Market efficiency can be viewed as an emergent property of a system whose state is determined by the interaction of multiple agents which compete to exploit the system’s inefficiencies, thereby making the system’s dynamics more random as predictability in those dynamics is exploited (and thereby reduced) until ideally it is no longer present. Liquidity in markets can be viewed as a form of systemic pressure on the system’s dynamics. More liquid markets are characterised by more agents and interactions which reduce noise in the dynamic process of the emergent variable (the price).

Market efficiency imposes the absence of temporal memory in price returns, but the presence of long memory in higher-order moments of returns and long-term dependence (autocorrelation) of absolute and squared returns have been observed and are now considered among the important stylised facts in markets [19]. For instance, in [20,21], later extended in [22], it was shown that order signs obey a long memory process, balanced by anti-correlated volumes which guarantee market efficiency. In financial time series analysis, through the generalised Hurst exponent analysis, it was observed that memory effects are related to the stage of maturity of the market, with more mature markets being more random [23]. However, these memory effects have been so far observed on the stochastic evolution of each single variable and not on the collective evolution of the dependency structure of the market.

With the present paper we provide the missing piece, connecting market structure and market memory by analysing the autocorrelation of market structures [24], through persistence of its filtered correlation matrix [25].

In this work, we indeed demonstrate that there exists a stable sub-structure in the system which is found via the higher order relation approach (i.e., beyond pairwise simplicial relations). We observe weak evidence that more efficient markets have more stable structures. We study how simplicial motifs—patterns of edges which define simplicial complexes on a graph [26,27], e.g., edges, triangles and tetrahedra—are persisting or decaying through the time evolution of the filtered networks computed over a rolling time window. In order to test the multivariate long memory properties of financial time series, we compare the motif persistence in networks from real data with motif persistence from a range of null models [28]—corresponding to a range of parsimonious assumptions on the underlying generative processes—for groups of time series. Each null model preserves different aspects of the time series, allowing to validate hypotheses about the long memory of market structures by ranking persistence decays of real time series against null models.

The persistence that we analyse in these systems can be compared with the autocorrelation in spin glasses. Indeed, in the physics of spin glasses, slow relaxation and correlation persistence have been studied for a very long time [29,30]. Simulation results and experiments reveal that the dynamic correlation function decays as a power law in the proximity and below the glass transition [31] in a similar way to what we report in the present work. In such systems one also observes aging effects which show an initial exponentially fast decay of the spin–spin autocorrelation function and then a freeze into slow dynamics [29]. Intriguingly, this was observed also in simulated network systems [32] and in real systems in the present work.

Edge persistence is also studied in Network Science in the form of survival (hard persistence) [33,34,35,36] as well as in Econophysics [37,38,39,40,41]. Further, ref. [42] defines one step node persistence analogously to the definition for edges in the present work.

In the present work we aim to demonstrate that topological filtering is a better suited tool to identify multivariate persistent structures with respect to the thresholding methods. We indeed observe that quantile thresholding yields analogous results to planar filtering for edge persistence, but it fails to identify higher dimensional motifs generating instead highly localised and clustered structures.

Further, we seek to demonstrate that our findings have a practical application by introducing an unsupervised technique to identify groups of stocks which share strong fundamental price drivers. This technique can be of particular use in less traded markets, where identifying structures with shared fundamental price drivers might otherwise require in-depth knowledge of the companies.

The rest of the paper is structured as follows. Section 2 describes the data, methods and definitions used for this work, Section 3 outlines the main findings, Section 4 discusses these findings and Section 5 concludes the work with suggestions for future works.

## 2. Materials and Methods

In this section, we describe the data used in this work, followed by a description of the time series null models and the filtering techniques applied to the correlation matrices. We then describe methods used to study the persistence of motifs.

### 2.1. Data

We select the 100 most capitalised stocks from four stock markets: NYSE, Italy, Germany and Israel (400 stocks in total). These markets range from highly liquid and more developed ones such as the New York Stock Exchange and the Frankfurt Stock Exchange to less liquid markets such as the Italian Stock Exchange and the Tel Aviv Stock Exchange.

We investigate daily closing price data from Bloomberg for:

- New York Stock Exchange (3 January 2014–31 December 2018);
- Frankfurt Stock Exchange (3 January 2014–28 December 2018);
- Borsa Italiana (Italian Stock Exchange) (3 January 2014–28 December 2018);
- Tel Aviv Stock Exchange (5 January 2014–1 January 2019).

The data includes 1258 daily prices observations for the NYSE, 1272 for FSE and BI and 1225 for TASE.

### 2.2. Time Series Null models

We generate ensembles of null models which preserve an increasing number of properties of the real time series.

#### 2.2.1. Random Return Shuffling

Individual stock log-return ($r_{t}=\log Price_{t}-\log Price_{t-1}$) time series are randomly shuffled, i.e., a random permutation along the time dimension of each variable is applied, to obtain a null model for noisy, spurious correlations. This model maintains the overall statistics of the values of each time series but eliminates any correlation structure.

#### 2.2.2. Rolling Univariate Gaussian Generator

We calculate the rolling mean $\mu_{t-\delta_{t},t}$ and standard deviation $\sigma_{t-\delta_{t},t}$ of the log-return series over the time window $\left(t-\delta_{t},t\right]$ for each node (stock) separately. We then generate ensembles by sampling the return $r_{t}$ at each point in time from the (rolling) univariate Gaussian distributions with sample mean and standard deviation $r_{t}∼N\left(\mu_{t-\delta_{t},t},\;\sigma_{t-\delta_{t},t}^{2}\right)$, with $N(\mu ,\;\sigma^{2})$ being a normal distribution with mean μ and standard deviation σ. This intends to simulate the process as a simple moving average with uncorrelated time-varying Gaussian random noise.

#### 2.2.3. Stable Multivariate Gaussian Generator

We calculate the mean μ (for each node) and covariance matrix Σ throughout the whole length of the log-return time series. We then generate ensembles by sampling the vector of returns $r_{t}$ at each point in time for all stocks from the fixed multivariate Gaussian with empirical means and covariance matrix, $r_{t}∼N\left(\mu ,\;\Sigma \right)$. This intends to represent an underlying fixed market structure with sampling noise.

#### 2.2.4. Rolling Multivariate Gaussian Generator

After obtaining the log-return time series, we calculate the rolling mean $\mu_{t-\delta_{t},t}$ (for each node) and covariance matrix $\Sigma_{t-\delta_{t},t}$ between the series. We generate ensembles by sampling the return at each point in time $r_{t}$ for all stocks from the (rolling) multivariate Gaussian distributions with sample means and covariance matrices $r_{t}=N\left(\mu_{t-\delta_{t},t},\;\Sigma_{t-\delta_{t},t}\right)$. This intends to detect the changing market structure and simulate the process as being generated by a multivariate Gaussian distribution with time-varying constraints on structural relations.

### 2.3. Correlation Matrix Estimation

We then compute for the time series correlation matrices with exponential smoothing from rolling windows of δ=126 trading days -corresponding to half a year- with smoothing factor of θ=46 days, following for an easy comparison the choices in [43]. This is done for all realisations of each null model ensemble and for the real data.

Correlations are noisy measures of co-movements of financial asset prices, which are often non-stationary within the observation window. Longer time windows benefit the measure’s stability, as we have more observations to estimate the N(N−1)/2 parameters of the matrix of *N* assets. However, a longer observation window can come with the disadvantage of weighting more and less recent co-movements equally with the risk of averaging over a period in which the values are non-stationary. In order to compensate for this effect, we apply the exponential smoothing method for Kendall correlations [43]. This yields to more stable correlations, as the method applies an exponential weighting to the correlation window, prioritising more recently observed co-movements.

### 2.4. Filtering: Quantile Thresholding and TMFG

We apply two filtering techniques with fundamental differences. The first filtering method is quantile thresholding, which corresponds to hard thresholding to generate an adjacency matrix through the binarisation of individual correlations. For a correlation value $v_{q}$ corresponding to the quantile level *q* of the matrix values, the adjacency matrix is defined as

$$A_{i,j}=\left\{\begin{array}{cc} \rho_{i,j}\geq v_{q}, & 1\\ \rho_{i,j}<v_{q}, & 0. \end{array}\right.$$

where $\rho_{i,j}$ is the estimated correlation matrix from the different models and from the real data. This filtering technique is entirely value-based with no structural or other constraints. We apply it by providing a quantile level *q* which yields edge sparsity analogous to that of the corresponding TMFG filter.

The second filtering technique is the TMFG method [17]. This topological filtering technique embeds the matrix with topological constraints on planarity in a graph composed of simplicial triangular and tetrahedral cliques. Edges are added in a constrained fashion with priority according to their (absolute) value. The graph essentially corresponds to tiling a surface of genus 0. This technique represents a filtering method that accounts for values, but also imposes an underlying chordal structural form which might help regularising the filtered graph also for probabilistic modeling [44]. Furthermore, this technique imposes higher order structures, namely triangles and tetrahedra, which are known to be a feature of financial markets and social networks.

### 2.5. Simplicial Persistence

We focus on temporal persistence of tetrahedral and triangular simplicial complexes (motifs) in the TMFGs and graphs filtered via quantile thesholding constructed from correlations over rolling windows. TMFG networks can be viewed as trees of tetrahedral (maximal) cliques connected by triangular faces, these are triangular cliques with different meaning in the taxonomy, called separators. If removed, separators split the graph into two parts. Not all triangular faces of the tetrahedral cliques are separators and we will refer to those which are not as triangles.

This distinction is discarded for the results in Section 3.2 in order to account for all triangles in the filtered graph, as quantile thresholding does not distinguish between triangular faces and separators.

**Figure 1:**
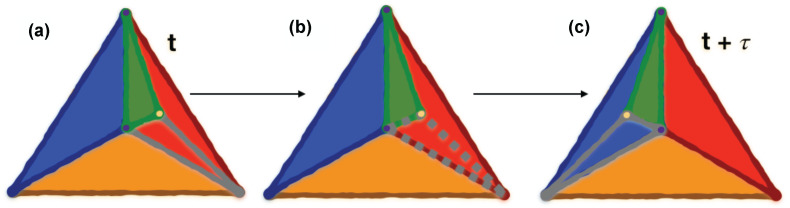
Motif persistence visualisation. (Color online) Visual representation of a TMFG structure’s motif (triangle) persistence in time. The green triangle in (**a**) is persistent through (**c**), while other two triangles (present in (**a**) within the red triangle) do not persist due to the rewiring of an edge. (**b**) shows one of non-persistent triangles with dashed contour. The rewired edge is also dashed and colors are used for visualisation purposes to “track” the triangles through the rewiring. This visualisation aims at showing the impact of edge rewiring on motif persistence and the difference between edge and motif persistence.

A motif corresponding to clique $X_{c}$ is considered soft-persistent at time t+τ if and only if the motif is present at both the initial time *t* and at t+τ. A visual intuition for motif (triangle) persistence through time is provided in Figure 1.

We investigate the decay in the number of persistent motifs between filtered correlation networks with observation windows progressively shifted by one trading day and we quantify how the average persistence decays with the time shift τ.

Here we use a form of soft persistence which is different from hard persistence (survival) of motifs which is more common in the literature [45,46]. In hard persistence a motif is found in a whole sequence of estimated networks without fail, in soft persistence we just require the motif to be found both at the beginning and at the end of a time interval. If we consider a generic motif *c*, defined as a specific collection of edges, and *C* as the set of all the partitions of the edges in a graph. The binary persistence value of motif c∈C at time *t* and t+τ is

(1) $$\begin{array}{c} P_{m}\left(X_{c}^{t,t+\tau }\right)=\left(X_{c}\in X_{C}^{t}\right)\wedge \left(X_{c}\in X_{C}^{t+\tau }\right). \end{array}$$

The average persistence for the entire clique set over *T* starting points at time shift τ is

(2) $$\begin{array}{c} {\langle P_{m}\left(X_{c}^{\tau }\right)\rangle }_{T,C}=\frac{1}{T}\cdot \frac{1}{\left|C\right|}\cdot \sum_{t=0}^{T}\sum_{c\in C}P_{m}\left(X_{c}^{t,t+\tau }\right). \end{array}$$

Where, considering the motif sets $X_{C}^{t}={\left\{X_{i}^{t}\right\}}_{i=c_{1},\cdots ,c_{\left|C\right|}}$ and $X_{C}^{t+\tau }={\left\{X_{i}^{t+\tau }\right\}}_{i=c_{1},\cdots ,c_{\left|C\right|}}$.

We observe that persistence decays as a power law with two regimes: one with a faster decay followed by one with a slower decay. The transition point between these two regimes, $\tau_{plat}$, is computed by minimising the unweighted average mean squared error (MSE) between the two power law fits over all possible transition points in time. The average motif persistence in the plateau regime is defined as

(3) $$\begin{array}{c} {\langle P_{m}\left(X_{c}\right)\rangle }_{T,T}=\frac{1}{T}\cdot \frac{1}{T-\tau_{plat}}\cdot \sum_{t=0}^{T}\sum_{\tau =\tau_{plat}}^{T}P_{m}\left(X_{c}^{t,t+\tau }\right). \end{array}$$

In order to verify that the persistence decay of motifs is not simply the consequence of the persistence decay of individual edges, we test the null hypothesis that motifs are formed by edges in the network whose existence is not mutually dependent. The assumption would imply that motif structures have no extra persistence beyond the individual edges that form them. The hypothesis being tested implies that motif persistence is simply the result of persistence characterising their component edges $\left(c_{1},c_{2},c_{3}\right)$:

(4) $$\begin{array}{c} P_{m}\left(\chi_{c}^{t,t+\tau }\right)=P_{m}\left(\chi_{c_{1}}^{t,t+\tau }\right)\cdot P_{m}\left(\chi_{c_{2}}^{t,t+\tau }\right)\cdot P_{m}\left(\chi_{c_{3}}^{t,t+\tau }\right), \end{array}$$

We also compare the decay exponents for multiple random stock selections over different markets to identify whether the steepness of motif decay (edge, closed triad or tetrahedron clique) is indicative of market stability/development stage. We further investigate more liquid markets such as the NYSE from both a quantitative and qualitative point of view.We classify motifs in the plateau by their soft persistence and study the sector structure of the most persistent motifs.

In order to provide an application to systemic risk (which can be quantified from the aggregate volatility in the system), we construct a portfolio containing all stocks in the ten most persistent motifs in the plateau region, as defined in Equation (3) (for each market). We then compare its volatility with that of random portfolios with the same number of assets.

## 3. Results

The main findings of this work are described in this section, starting with an overview of results on the long memory of edges and simplicial complexes in TMFG-filtered correlation networks. The section continues with an analysis of null models of financial market structures, described in Section 2.2, and a comparison with real data to gain insights about the generative process of the stochastic structure. We then suggests how soft persistence captures the underlying change in market structure by relating its decay exponent to the efficiency (a proxy for stability) or average traded volume in the market (a proxy for fluidity which yields well-defined stable structures). We conclude the section with results in systemic risk applications to financial portfolios where we show that the most persistent motifs correspond to stocks in the same sector and demonstrate how the portfolio of 10 most persistent motifs is highly volatile and systemic.

### 3.1. Long-Term Memory of Motif Structures

**Figure 2:**
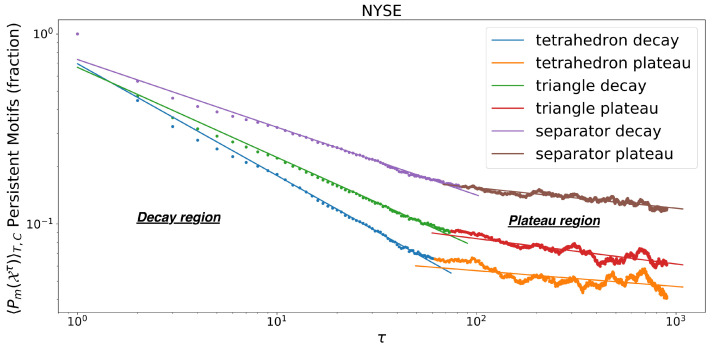
Persistence Decay. Decay of triangular clique faces, separators and clique motifs persistence for 100 NYSE stocks, as a function of time interval τ=[0,900] (average over 200 starting points). The motifs and network are obtained via TMFG filtering. The two power-law regimes are identified by the minimum MSE sum of the fits. The second region in termed “plateau” in contrast with the strong decay observed in the “decay” region although we recongnise that.

The plot in Figure 2 shows the power law decay (evident from the linear trend in log-log scale) in ${\langle P_{m}\left(X^{\tau }\right)\rangle }_{T=200,C}$ vs. τ, followed by a plateau region that also decays as a power law, but with a smaller exponent. We also observe that all motif decays have $\tau_{plat}\in \left[\delta t_{window}/2,\delta t_{window}\right]$, where $\delta t_{window}$ represents the length of the estimation window of the correlation matrix. The window used has $\delta t_{window}=126$ trading days and a value of θ=46 for exponential smoothing, as per [43]. The choice of $\delta t_{window}$ corresponds to roughly 6 months of trading and satisfies $N<\delta t_{window}$, with *N* the number of assets in the correlation matrix. The correlation matrix is hence well-conditioned and invertible. On the other hand the exponential smoothing with θ=46 mainly considers recent observations from the latest few months.

There are N−3=97 tetrahedral cliques in the starting TMFG networks and 3N−8=292 face triangles.

In Figure 2 we notice that the minimum MSE for the two linear fits is achieved at the transition point between the decay phase and the plateau. The transition point $\tau_{plat}$ can therefore be identified by minimising a standard fit measure with two phases, which strengthens the unsupervised nature of our method. The method for minimum MSE search is described Section 2.5.

### 3.2. Null Models of Persistence in Filtered Structures

We report results for the edge and motif (triangle) persistence for real data as well as for the null models described in Section 2.2. We compare real data with null models and TMFG filtering with quantile thresholding.

**Figure 3:**
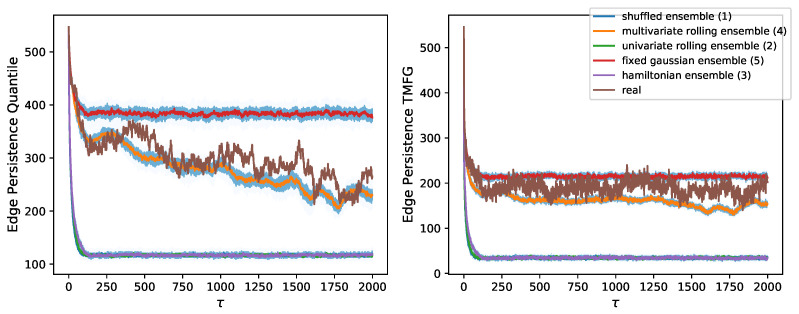
Decay of number of persistent edges in null models. Decay in the number of persistent edges with $\delta_{\tau }=126$ for the time series null models of market returns and real data for the NYSE. We notice how for both TMFG filtering and quantile thresholding the real data lies between the rolling multivariate Gaussian ensemble and the stable multivariate Gaussian ensemble. This indicates that the real market structure does evolve slowly in time, but with persistence beyond what can be inferred from estimates of its covariance structure. Here we plot the number of persistent edges instead of persistence as a probability to show that both filtering methods start from the same edge sparsity.

Figure 3 shows the decay in edge persistence for both filtering methods. We notice that the random shuffling null model lies at the bottom, as it should produce completely random structures with little residual persistence due to probabilistic combinatorics and structural filtering constraints in the TMFG. This shows that persistence is not an artifact of any of the filtering techniques used and not a mere result of return volatility of individual assets (which is preserved by return shuffling). From Figure 3 we also notice that the rolling univariate Gaussian model lies just above as it does not account for structure at all and only preserves rolling means and standard deviations, this shows how persistence cannot merely be attributed to common long term trends or volatility variations. This null model carries some broad sense of structure and market direction and it shows how persistence does not merely originate from overall market trends. We then find a second cluster, of structured models, with the rolling multivariate Gaussian at the bottom. This shows how market persistence goes beyond asset means and covariance, even after spurious structures have been removed. We then find the real data, just below the stable multivariate Gaussian. This shows how markets have slowly evolving structures.

**Figure 4:**
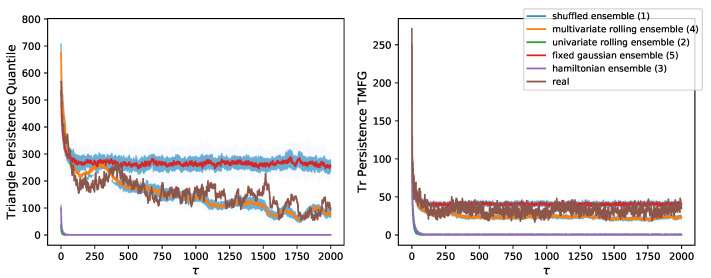
Decay of number of persistent motifs (triangle) in null models. Decay in the number of persistent motifs with $\delta_{\tau }=126$ for the time series null models of market returns and real data for the NYSE. We notice how for TMFG filtering the real data still lies between the rolling multivariate Gaussian ensemble and the stable multivariate Gaussian ensemble (as in Figure 3). We instead notice that the decay ordering is noisier for quantile thresholding, showing how the method’s focus on individual connections affects it generalisation to motifs. This is despite the higher number of motifs in the quantile thresholding graph. Here we plot the number of persistent motifs instead of persistence as a probability to show that in spite of both filtering methods starting from the same edge sparsity (Figure 3) the number of initial cliques and persistent cliques is very different across methods. This is likely due to the highly clustered structure of thresholding-based networks.

Figure 4 shows the decay in triangular motif persistence for both filtering methods. We notice results analogous to those in Figure 3 for TMFG filtered graphs. Graphs filtered through quantile thresholding instead show a high level of noise in their top cluster (where structure is present). A higher number of motifs than those of the TMFG is found, but the ranking of null models is at times inconsistent, as well as the position of the decay curve for real data. We would have expected some triangles to break when looking at edge persistence only, as well as to find that the clustering coefficient decreases in persistent graphs (as it does in TMFG graphs). The clustering coefficient for quantile thresholding-persistent graphs is also found to be much higher, suggesting that the filtered structure is highly localised and clustered, while that of the TMFG is more distributed, identifying systemic groups of stocks throughout the market structure.

### 3.3. Market Classification via Decay Exponent

We now consider how the decay exponent of TMFG graphs behaves across markets. Table 1 compares the decay exponents for cliques (4-cliques), triangular motifs and clique separators in the NYSE, German stock market, Italian stock market and Israeli stock market. The decay exponent α is obtained from the power law decay fit:

(5) $$\begin{array}{c} {\langle P_{m}\left(X^{\tau }\right)\rangle }_{T,C}=\beta \cdot \tau^{\alpha } \end{array}$$

We notice from the results in Table 1 that the NYSE, which is clearly the most developed and liquid stock market, has the lowest decay exponent (in modulus, which corresponds to the slowest decay) for both cliques and triangles. This indicates that its correlations are more stable on a shorter time window. Germany and Italy have similar values for clique exponents, with Germany seemingly more stable in terms of triangular motifs. Israel, a younger and less liquid stock market, follows with a faster decay in both tetrahedral cliques and triangular motifs. The ordering of these markets is not clearly identifiable in clique separators as noise in the data does not allow for the two decay regimes to be correctly identified in all markets (in this case for Italy). Separators have a distinct role and meaning in the graph’s taxonomy and further work should allow for a more thorough analysis of those.

We observe promising results for a monotically increasing relation between the decay exponent and the average daily volume of the market. The solidity of this result shall be investigated in future works.

In Table 1 the decay exponent is not adjusted by the probability that all edges in the clique must be present in the temporal layer for the clique to exist. We show in Table 2 that, when adjusted by the probability of all its edges existing simultaneously, triangular motifs have a slower decay than individual edges. The results in Table 2 are obtained from a set of randomly selected stocks different to those used for Table 1. This adds further confidence in the results and their generality.

We stress that Table 2 falsifies the hypothesis that motifs are formed by edges in the network whose existence is not mutually dependent (Equation (4)). This is falsified by the consistently lower decay exponent (in modulus) for adjusted persistence of triangular motifs. We can then conclude that motifs are more stable structures across temporal layers of the network, with significant interdependencies in their edges’ existence.

### 3.4. Sector Analysis in Persistent Motifs

**Figure 5:**
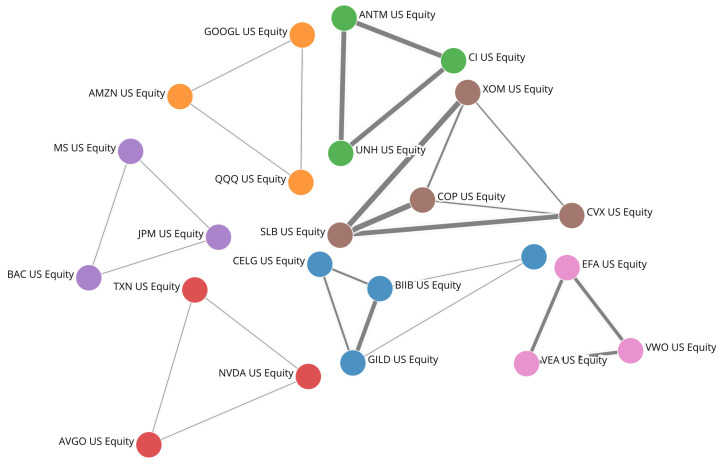
Persistent NYSE motifs visualised. Network representation of the ten most persistent triangular motifs in the TMFG layers for the 100 most capitalised stocks of the NYSE.

Figure 5 provides a visualisation of the network components formed by the ten most persistent triangles in the NYSE. We observe that all strongly persistent triangles have elements which belong to the same industry sector. Table 3 shows this for the same ten triangles displayed in Figure 5. We notice that stock prices in the sectors in Table 3 are mostly driven by sector-wide fundamentals, which justify the persistent structure in the long term Figure 5. Other motifs are constituted by ETFs and their main holdings (The reason for the existence of these motifs is intuitive and does not affect our analysis, as ETF-related motifs are unlikely to be present in the network formed by a random selection of stocks or by stocks in a portfolio. These motifs are present here as we focus on the 100 most capitalised stocks in the NYSE, which include ETFs).

We also investigate whether motif persistence and motif structures can be easily retrieved from the original correlation matrix. The purpose of this is to check that our TMFG filtering method is not redundant and trivially replaceable. To test this, we consider the ten most present persistent triangles across the plateau region and check their overlap with the ten most correlated triplets in each unfiltered correlation matrix. We find that no more than one triangle lies in the intersection between the two sets, in each temporal layer. We also check the correlation between motif persistence and the average sum or product (results are equivalent for our purpose) of its individual edges’ correlation for all unfiltered correlation layers. We observed through the Pearson and Kendall correlation values that the two measures are only loosely related, as correlation explained no more than 20% of the variance in the set of variables with large persistence.

### 3.5. Portfolio Volatility and Systemic Risk of Persistent Motifs vs. Random Portfolios

We check that a subgraph (a portfolio, i.e., group of stocks) formed by the 10 most persistent motifs in each market has a highly enhanced out of sample standard deviation $\sigma_{sub}$ (volatility—standard deviation of the mean log-return of the subgraph elements’ prices) due to its stable correlations.

To do this, we consider the $\sigma_{sub}$ of the motif subgraph and a distribution of $\sigma_{sub}$ for $10^{5}$ randomly selected subgraphs with the same number of nodes (stocks).

As expected, we observe that the persistent motif subgraph is characterised by a $\sigma_{sub}$ over two standard deviations above the mean of the distribution as well as above its 75% quantile throughout the considered markets. We should highlight that the $\sigma_{sub}$ of subgraphs is evaluated out of sample with respect to the period the persistence was calculated on, showing that this method is not only observational, but also predictive.

Due to the more theoretical nature of this work, we refer the interested reader the work by some of the authors of this paper for a more thorough analysis of subgraph applications and forecasting [47].

## 4. Discussion

The power law decay of edge and simplicial motifs persistence, reported in Figure 2, suggests that market structures are characterised by a slow evolution with long memory. This decay type is in contrast with an exponential decay of the persistence which would imply instead short or no memory in the system. This observation is in line with the works by Bouchaud et al. and Lillo at al. in [20,22,23,48], where power law decays in autocorrelation are identified as manifestations of long-memory processes in efficient markets. However, it extends the concept to higher order structures.

The comparison between soft persistence in correlation structures from real data and artificial data generated from different null models (Figure 3 and Figure 4) demonstrates that the persistence of real structures goes beyond all univariate null models, hence confirming long memory as a characteristic requiring structural constraints. Additionally, we demonstrate that real structures overcome the persistence of the rolling multivariate Gaussian, suggesting that pairwise covariances and moving averages do not suffice to induce the long memory present in real markets. As per the analysis on motif persistence beyond that of individual edges, we suggest that higher order relations in terms of structural evolution are present. The ordering of null models in Figure 4 further supports the validity of the persistence measure.

The comparison of simplicial persistence of triangles between quantile thresholding and TMFG filtered graphs, reported in Figure 4, reveals that quantile thresholding struggles to separate the decay of real structures from that of rolling Gaussian generated ones. This could be attributed to the “local” nature of the method, which matches the pairwise interpretation of relations generated from a rolling Gaussian. TMFG filtered graphs instead, perhaps due to their non-local embedding, provide a consistent ordering of null models with relatively low noise.

The ability to correctly identify persistent motifs throughout the market sample is essential as the most persistent motifs were found to be highly systemic (Section 3.5). Persistent structures in quantile thresholded graphs present higher and more stable clustering coefficients. This suggests a very localised and compact structure. TMFG filtered graphs instead present a lower clustering coefficient and a slower decay with τ, as expected since some structures break. This is further evidence of the ability of the TMFG filtering method to identify meaningful persistent structures throughout the market. The issue with quantile thresholding is likely due to the method being merely value-based with no sensible structural constraint, differently from the TMFG.

The ranking of national markets based on their decay exponents in Table 1 can be interpreted in terms of the reduction of estimation noise in more liquid markets, as large deviations become less likely and correlations as well as prices more reflective of the underlying generative processes and structures. Structures are perhaps clearer too and deviations are exploited more quickly if they emerge. This suggests that more efficient and capitalised markets are characterised by structures which are more stable in time and better reflected by the data. The decay exponent ranking also leads to the conclusion that more developed markets are characterised by more meaningful underlying structures and cliques, suggesting that systemic risk may represent a greater threat in developed markets.

The results in Table 2 support the hypothesis that motifs constitute meaningful structures in markets, beyond their individual edges. These results test the independence null model of individual edges in motif formation and show solid evidence to reject it. We can then conclude that highly persistent motifs are not a mere consequence of highly persistent individual edges, but also of the correlation in those edges existing concurrently. This results ties in with the above discussion on the issues with locality of filtering methods and generative processes.

Table 3 strengthens the importance of persistent motifs. Indeed, the ten most persistent motifs visualised in Figure 5 are representative of industry sectors in the NYSE. These sectors are not identified by the motifs with higher edge correlation, which instead are dominated by motifs often due to correlation noise in high volatility stocks. Persistence and the identification of persistent motifs are hence found to be non-trivial with respect to correlation strength of individual edges or motifs. The impact on portfolio diversification (reduction in the variability $\sigma_{sub}$) of the motifs in Figure 5 indicates that these structures are highly relevant for systemic risk and portfolio volatility, with high predictive power provided by the long memory property of persistence, which is an intrinsic temporal feature. As these motifs are not characterised by noticeably strong correlations, a common variance optimisation of the portfolio is unlikely to optimise the weights to sufficiently minimise the risk from these highly systemic structures.

The systemic relevance of persistent motifs as well as their out of sample forecasting power are shown by the results in Section 3.5 and in [47], where significantly higher out of sample portfolio volatility is observed for the subgraph of persistent motifs. The motif subgraph $\sigma_{sub}$ is significantly above both the mean and median of the random subgraphs’ $\sigma_{sub}$ distribution.

This is an example of how just selecting nodes (stocks) from the ten most persistent motifs forms a subgraph with higher long term variability $\sigma_{sub}$. Clearly when aiming for a reduction in systemic risk, low $\sigma_{sub}$ (the opposite) is the objective. The observations from Section 3.5 and [47] lay the ground for the construction of portfolios where asset weights aim to reduce the volatility originating from persistent correlations in motif structures.

## 5. Conclusions

The present work introduces the concept of simplicial persistence, focusing on the soft persistence in simplicial cliques. This measure is applied to a complex system with a slowly evolving stochastic structure, namely financial markets. The graph structures are obtained from Kendall correlations with exponential smoothing and filtered with the TMFG or through quantile thresholding. The slow evolution of these systems with time manifests long memory in their structure with a two regime power law decay in persistence with time. The transition point between regimes is identified in an unsupervised way with mean-squared error minimisation.

Null models of market structure are then used to test hypotheses about the generative process underlying the system. Two persistence decay clusters are observed, where the least persistent corresponds to null models with no structural constraints and the upper one (most persistent) comprises the rolling multivariate Gaussian (lowest), real data, and the stable multivariate Gaussian (highest).

Decay exponents for different markets are then observed to provide a ranking corresponding to their efficiency, which suggests that, despite these systems being less predictable in their individual series, they are more stable and predictable in terms of structure. Most persistent motifs are found to correspond to sectors where the price of stocks is mostly driven by sector-wide fundamentals.

Based on the ability of simplicial persistence to forecast and identify strongly correlated clusters of stocks, the impact of persistence-based systemic risk on portfolio volatility is verified with a comparison between the ten most persistent motifs portfolio and random portfolios of the same size [47]. Simplicial persistence of higher order structures in real data and null models is hardly recognised by value-based thresholding methods which are unable to identify persistent cliques throughout the market sample.

The present work provides further evidence of how network analysis and complex systems can enhance our understanding of real world systems beyond traditional methods. Our results and methods lay the ground for future studies and modelling of the evolution of stochastic structures with long memory.

## Figures and Tables

**Table 1:** Exponents for the decay power law regime computed with MSE.The analysis refers to 100 randomly selected stocks amongst the 500 most capitalised, over time intervals $\tau = [0,900)$ and $t= [0,\cdots ,200)$ different initial temporal network layers. For all motif analyses in this work, triangles and separators constitute non-overlapping sets, as these represent theoretically and taxonomically different structures and decay characteristics.

Market	Clique	Triangular Motif	Clique Separator
NYSE	−0.392	−0.493	−0.245
Germany	−0.792	−0.598	−0.381
Italy	−0.785	−0.811	−0.174 *
Israel	−1.024	−0.866	−0.728

* Result compromised by regimes not well identified for motif decay in large systems (≈100 stocks).

**Table 2:** Exponent for the power law decay regime identified by MSE in different sample markets. The analysis refers to 100 randomly selected stocks amongst the 500 most capitalised, over time intervals $\tau = [0,900]$ and $t= [0,\cdots ,200]$ different initial temporal network layers.

Market	Edge	Triangular Motif	Triangular Motif **
NYSE	−0.164	−0.398	−0.133
Germany	−0.265	−0.471	−0.157
Italy	−0.144 *	−0.458	−0.153
Israel	−0.397	−0.830	−0.277

* Result compromised by regimes not well identified for edge decay in large systems (≈100 stocks); ** Motif exponent adjusted by the probability of simultaneous edge persistence in the motif.

**Table 3:** Motif components and Financial Times sector affiliation for the ten most persistent motifs in the NYSE’s 100 most capitalised stocks.

Node 1	Node 2	Node 3	FT Sector
Biogen Inc.	Gilead Sciences Inc.	Celgene Corp	Biopharmaceutical
UnitedHealth Group Inc.	Cigna Corp	Anthem Inc.	Health Care
Biogen Inc.	Gilead Sciences Inc.	Amgen Inc.	Biopharma/tech
Bank of America Corp	JPMorgan Chase & Co	Morgan Stanley	Financials-Banks
Vanguard FTSE ETF **	MSCI EAFE ETF	Vanguard FTSE ETF ***	Index ETFs
Invesco QQQ Trust *	Amazon.com Inc	Alphabet Inc	Tech
ConocoPhillips	Schlumberger NV	Exxon Mobil Corp	Oil & Gas
NVIDIA Corp	Texas Instruments Inc.	Broadcom Inc.	Tech Hardware
Chevron Corp	Schlumberger NV	Exxon Mobil Corp	Oil & Gas
Chevron Corp	ConocoPhillips	Schlumberger NV	Oil & Gas

* ETF on NASDAQ—Top Holdings include Amazon, Facebook, Apple, Alphabet; ** Vanguard FTSE Developed Markets Index Fund ETF Shares; *** Vanguard FTSE Emerging Markets Index Fund ETF Shares.

## Data Availability

The data presented in this study are available on request from the corresponding author. The data are not publicly available as it was obtained from Bloomberg LP and are available on their website/platform.
